# DNA phosphorothioation in *Streptomyces lividans*: mutational analysis of the *dnd *locus

**DOI:** 10.1186/1471-2180-9-41

**Published:** 2009-02-20

**Authors:** Tiegang Xu, Jingdan Liang, Shi Chen, Lianrong Wang, Xinyi He, Delin You, Zhijun Wang, Aiying Li, Zhongli Xu, Xiufen Zhou, Zixin Deng

**Affiliations:** 1 Laboratory of Microbial Metabolism, and School of Life Sciences & Biotechnology, Shanghai Jiaotong University, Shanghai 200030, PR China

## Abstract

**Background:**

A novel DNA phosphorothioate modification (DNA sulfur modification), in which one of the non-bridging oxygen atoms in the phosphodiester bond linking DNA nucleotides is exchanged by sulphur, was found to be genetically determined by *dnd *or *dnd*-counterpart loci in a wide spectrum of bacteria from diverse habitats. A detailed mutational analysis of the individual genes within the *dnd *locus in *Streptomyces lividans *responsible for DNA phosphorothioation was performed and is described here. It should be of great help for the mechanistic study of this intriguing system.

**Results:**

A 6,665-bp DNA region carrying just five ORFs (*dndA-E*) was defined as the sole determinant for modification of the DNA backbone in *S. lividans *to form phosphorothioate. This provides a diagnostically reliable and easily assayable Dnd (DNA degradation) phenotype. While *dndA *is clearly transcribed independently, *dndB*-*E *constitute an operon, as revealed by RT-PCR analysis. An efficient mutation-integration-complementation system was developed to allow for detailed functional analysis of these *dnd *genes. The Dnd^- ^phenotype caused by specific in-frame deletion of the *dndA*, *C*, *D*, and *E *genes or the enhanced Dnd phenotype resulting from in-frame deletion of *dndB *could be restored by expression vectors carrying the corresponding *dnd *genes. Interestingly, overdosage of DndC or DndD, but not other Dnd proteins, *in vivo *was found to be detrimental to cell viability.

**Conclusion:**

DNA phosphorothioation is a multi-enzymatic and highly coordinated process controlled by five *dnd *genes. Overexpression of some proteins *in vivo *prevented growth of host strain, suggesting that expression of the gene cluster is strictly regulated in the native host.

## Background

Most of the commonly found structural changes in DNA are due to methylation of selected bases. In some viral DNAs, certain bases may be hydroxymethylated or glucosylated [1-3]. Altered or unusual bases in DNA molecules often have significant physiological implications, such as DNA replication control, gene regulation, or protection of the respective organisms from invasion by foreign DNA [4].

In contrast to other types of DNA modification, *S, lividans *has a site-specific and stereo-selective sulfur modification on the DNA backbone termed phosphorothioation [5-7]. This sulfur modification occurs specifically between two guanine nucleotides in *S.lividans *[6,8]. The sulfur-modified DNA suffers double-stranded cleavage at the modification sites during normal and pulsed-field gel electrophoresis [6,9-13]. The Dnd phenotype was proven to be a peracid-mediated, oxidative and amine-catalysed reaction to form a phosphorothioated DNA backbone [6,14,15]. The Dnd phenotype can be overcome by replacing Tris with Hepes in the electrophoresis buffer or by adding a certain concentration of thiourea to Tris-containing buffers [14,15].

In *S.lividans*, this DNA sulfur modification was found to be determined by a *dnd *gene cluster carrying five open reading frames (ORFs, *dndA-E*) [5]. Homologous *dnd *gene clusters and/or Dnd phenotypes are found in many strains of *Streptomyces, E. coli*, *Bacillus*, *Salmonella*, *Klebsiella, Enterobacter*, *Mycobacterium, Vibrio, Pseudomonas, Pseudoalteromonas, Hahella, Oceanobacter, Geobacter, Pelagibacter, Roseobacter, Mesorhizobium, Serratia*, *Acinetobacter*, and *Clostridium*, as well as in certain *Archaea *and unidentified marine microbes, indicating that DNA sulfur modification is a widespread phenomenon in prokaryotes [16].

Here we attribute DNA phosphorothioate modification to a *dnd *gene cluster consisting of a 6,665-bp region of DNA carrying just five genes. We confirmed by transcriptional analysis that *dndB-E *constitute an operon, and made systematic in-frame deletion mutations within each gene or combinations of the five *dnd *genes before performing a series of complementation analyses to evaluate the roles of individual *dnd *genes in DNA sulfur modification.

## Results

### Identification of a minimal *dnd *region

**Figure 1:**
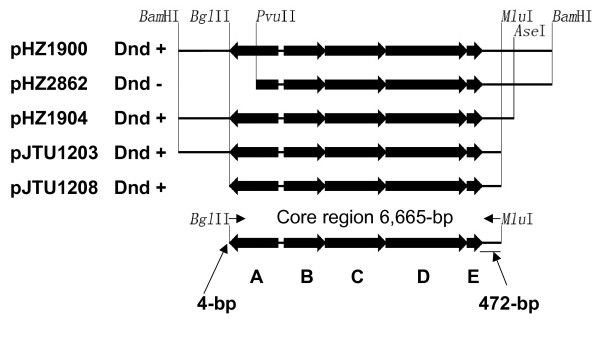
**Localization of the boundaries for *dnd *gene cluster**. pSET152-derivatives with the ability to confer Dnd (+ or -) phenotypes are indicated in line with their insert fragments. Five arrows from left to right represent five the ORFs of the *dnd *gene cluster (*dndA-E*). Directions of the arrows indicate the transcriptional directions of the genes.

In an effort to precisely localize the region responsible for the Dnd phenotype and obtain unambiguous evidence on the genes involved in DNA phosphorothioation, we made a series of pHZ1900 derivatives by removing end segments from a *ca*. 10-kb fragment of DNA carrying some likely *cis*-acting elements using convenient restriction sites, thus identifying a core region carrying only five *dnd *genes. A combination of restriction fragments (Fig. 1) was incorporated into appropriate sites of integrative vector pSET152 [17] to produce four plasmids (pHZ1904 [5], pJTU1203, pHZ2862, and pJTU1208). Mediated by the *attP *site of *Streptomyces *phage ØC31 present on pSET152, these vectors can site-specifically integrate into the *attB *site in the chromosome of *S. lividans *ZX1 [9] after transfer by conjugation from *E. coli *ET12567/pUZ8002 into ZX1. The DNA of these ZX1-derivative strains was either degraded (Dnd^+^) or stable (Dnd^-^) during electrophoresis (Fig. 1). The minimal *dnd *region conferring the Dnd phenotype (Dnd^+^) was localised to a 6,665-bp fragment on pJTU1208. The left and right borders of the minimal *dnd *cluster are only 4-bp and 472-bp from the stop codons of *dndA *and *dndE *(Fig. 1), respectively, confirming that five genes are necessary and sufficient for DNA phosphorothioation.

### Transcriptional analysis of the *dnd *genes

Bioinformatic analysis of the 6,665-bp region of pJTU1208 (GenBank accession number DQ075322) suggests that *dndA *and *dndB-E *are divergently transcribed. The facts that the 3' end of *dndB *and the 5' end of *dndC *overlap by 4 bp (ATGA, position 3,605 to 3,608), that the initiation codon (ATG) of *dndD *precedes the 3' end of *dndC *by 12 bp (5088-ATGCACCTGCATAA-5098), and that the initiation codon of *dndE *(ATG) is 9 bp upstream of the stop codon of *dndD *(ATGCCGTCTGA) strongly imply that the *dndB-E *might constitute an operon.

**Figure 2:**
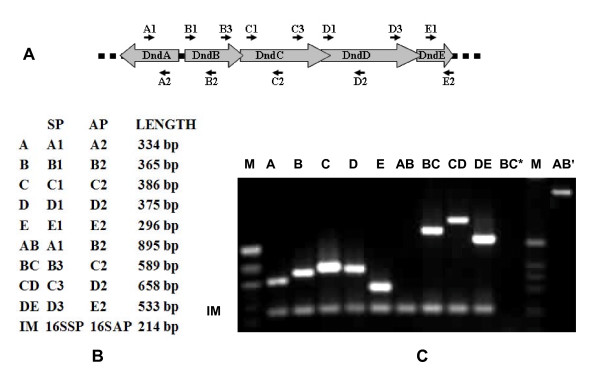
**RT-PCR analysis of the *dnd *genes transcripts**. *dnd *gene transcripts were reverse transcribed and amplified. (A) Relative positions and directions of corresponding primers are marked with black arrows. (B) Amplification products with sense primer (SP), anti sense primer (AP) and their corresponding lengths. Intra-*dnd *gene amplification products are indicated as *dnd *gene names, while products of regions between *dnd *genes are named linking two corresponding genes such as AB. Amplification of 16S rRNA is used as an internal control marker (IM). (C) Electrophoresis of RT-PCR products. The amplification products are labeled as in Figure 2B. Reverse transcriptase inactivation (BC*) and without DNase treatment (AB') were carried out as negative and positive controls. DNA markers are labeled as "M".

To prove divergent transcription of *dndA *and a hypothetical *dndB-E *operon, we performed a transcriptional analysis on the minimal *dnd *cluster by RT-PCR. RNA was extracted from *S. lividans *1326 and amplified by RT-PCR using oligonucleotide primers depicted in Fig. 2A. The PCR products were fractionated by electrophoresis (Fig. 2C). As an internal control, 16S rRNA was amplified in all samples. The appearance of DNA bands (Fig. 2C), which were amplified using different sets of primers (Fig. 2A and 2B), unambiguously suggests that *dndB-E *are co-transcribed as a single operon in *S. lividans *1326. The absence of DNA bands using primers A1 and B2 (Fig. 2C lane AB) suggests a lack of co-transcription in the region between A1 and B2, confirming independent transcription of *dndA *and *dndB-E*.

### A mutation-integration system for functional analysis of individual *dnd *genes

**Figure 3:**
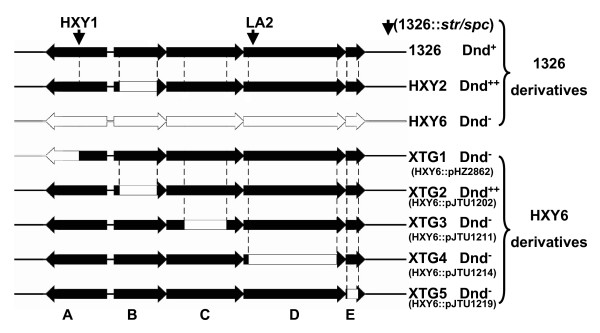
***dnd *mutants**. Black arrows represent *dnd *genes and their transcriptional directions. White blocks/arrows represent in-frame deletions in the corresponding genes.

As demonstrated by the transcriptional analysis, *dndB-E *constitute an operon. We therefore inactivated each of the five *dnd *genes independently to examine their effect on the Dnd phenotype in terms of DNA phosphorothioation. Early experiments on disruption of *dndA *(mutant HXY1) and *dndD *(mutant LA2) by a *str/spc *cassette clearly abolished the Dnd phenotype [5] (Fig. 3) but could not provide unambiguous evidence for the function of *dndD *as insertion of antibiotic resistant genes could block expression of downstream gene(s) of an operon by a polar effect.

We attempted to make in-frame deletions internal to individual *dnd *genes at their corresponding chromosomal loci to avoid polar effects. Apart from the *dndB *in-frame deletion mutant HXY2 [8] (Fig. 3), extensive efforts to obtain mutants specific to other *dnd *genes directly on the wild-type *S. lividans *1326 chromosome failed for unknown reasons. We therefore attempted to develop a mutation-integration system by first generating a complete set of in-frame deletions of individual *dnd *gene *in vitro *in *E. coli*. These mutated *dnd *genes were then integrated back into the chromosome of *S.lividans *HXY6 (generated by targeted deletion of the complete *dnd *locus, [8]). A complete set of pSET152-derived integration plasmids with targeted in-frame deletions of the five *dnd *genes was generated by PCR and cloned into *E. coli *[detailed in Methods, pHZ2862 (651-bp deletion in *dndA*); pJTU1202 (729-bp deletion in *dndB*); pJTU1211 (819-bp deletion in *dndC*); pJTU1214 (1,704-bp deletion in *dndD*); and pJTU1219 (216-bp deletion in *dndE*), respectively]. These plasmids were introduced into HXY6 to obtain mutants XTG1-XTG5 with in-frame deletions in *dndA*-*E *in a uniform parental background.

**Figure 4:**
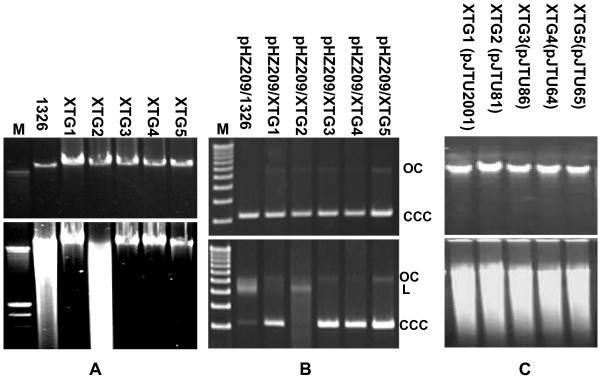
**Dnd phenotype of 1326 and related *dnd *mutants**. (A) Dnd phenotype of chromosomal DNA for 1326 and related *dnd *mutants. (B) Dnd phenotype of plasmids pHZ209 isolated from 1326 and related *dnd *mutants. (C) Dnd phenotype of chromosomal DNA from complemented *dnd *mutants. DNA was first treated with TAE (top panel) or peracetic acid TAE (bottom panel) before fractionation by electrophoresis in TAE with added thiourea. M: DNA markers; CCC: covalently closed circular plasmid; OC: open circular plasmid. L: linear plasmid.

Isogenic mutant strains (XTG1-XTG5) were assayed for their Dnd phenotype. Interestingly, while the Dnd phenotype, as displayed by degradation of chromosomal (Fig. 4A) or plasmid pHZ209 (Fig. 4B) DNA isolated from strains XTG1, XTG3, XTG4, and XTG5 (corresponding to *dndA, C, D, E*) was clearly abolished, DNA isolated from XTG2 retained the Dnd phenotype, clearly showing that *dndA*, *C*, *D*, and *E *are all essential for DNA phosphorothioation. Single-stranded DNA modification, which should be indicated by shifting of the covalently closed circular (CCC) to the open circular (OC) form for plasmid pHZ209 DNA if cleaved by the electrophoresis buffer, was not observed with these mutants (Fig. 4B), as also found for HXY1 (data not shown).

A close comparison of the Dnd phenotypes displayed by the wild-type 1326 and the *dndB *mutant XTG2, however, revealed a clear difference. The degradation "smear" from the genomic DNA of XTG2 migrated much faster than that from wild-type strain 1326 (Fig. 4A). Smaller genomic DNA fragments, or more frequently degraded genomic DNAs, were observed in the mutant XTG2 than the wild-type strain 1326. In other words, the *dndB *mutation appeared to aggravate the Dnd phenotype, in agreement with the enhanced Dnd phenotype of another in-frame deletion mutant HXY2 [8]. On the other hand, degradation of the circular plasmid pHZ209, as shown by the relative intensities of the linearized pHZ209, appeared to be more intense from XTG2 than from 1326. Almost all the circular plasmid pHZ209 from XTG2 was degraded as linearized forms, but only about two-thirds of the circular plasmid pHZ209 from 1326 was linearized (Fig. 4B).

### Rescue of the Dnd phenotype of *dnd *mutants by complementation

The first direct evidence that the Dnd phenotype, reflecting DNA phosphorothioation, involves the combined action of five independent proteins (DndA-E) comes from complementation experiments using plasmids expressing individual Dnd proteins. This was achieved by the construction of individual *dnd *gene expression plasmids using pHZ1272 [18], an *E. coli-Streptomyces *shuttle expression vector derived from pIJ6021 with a strong thiostrepton-inducible *P*_*tipA *_promoter [19]. Firstly, DNA fragments carrying individual *dndA-E *genes were cloned in-frame into pHZ1272 to generate expression plasmids (pJTU2001, carrying *dndA*; pJTU81, carrying *dndB*; pJTU86, carrying *dndC*; pJTU64, carrying *dndD*; and pJTU65, carrying *dndE*). Secondly, the expression plasmids were independently introduced by transformation into the corresponding mutant strains XTG1, 2, 3, 4, and 5 (with in-frame-deletions of *dndA*, *B*, *C*, *D*, and *E*, respectively). Even without induction of the *P*_*tipA *_promoter by addition of thiostrepton, strains XTG1, 3, 4, 5 carrying their counterpart expression plasmids recovered the Dnd phenotype of the wild-type strain 1326 (Dnd^+^), while XTG2 carrying pJTU81 (with a complete *dndB *gene) abolished enhanced Dnd phenotype (Dnd^+^) with recovery of the original Dnd phenotype (Dnd^+^) comparable with that of the wild-type strain 1326 (Fig. 4C).

**Figure 5:**
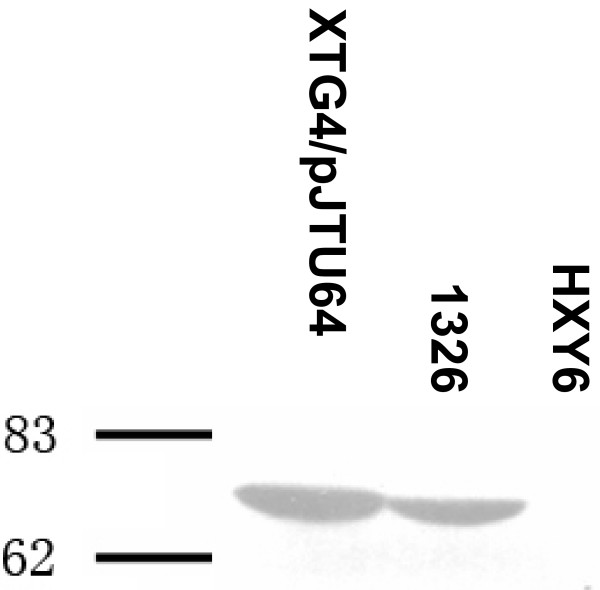
**Western blotting for detecting expression of Dnd proteins in *S. lividans *1326 and derivative strains**. Rabbit polyclonal antibody to DndD reacted with the protein extracted from wild-type *S. lividans *1326 or strain XTG4/pJTU64 (a pHZ1272-derived *dndD *expression vector).

As additional evidence, we cloned *dndD *into pET15b to obtain an expression plasmid (pHZ2893) for the production of an N-terminal His-tag fusion protein. The purified DndD protein was then used for the production of rabbit anti-DndD polyclonal antibody. When we used this antibody to detect native DndD protein expression, we observed identical bands with a size of 74.6 KD in the expression strain XTG4/pJTU64, and wild-type *S. lividans *1326 (Fig. 5). As a negative control, a 1326 derivative with complete deletion of the *dnd *gene cluster (HXY6) produced no signal in the corresponding position (Fig. 5). The protein size agrees well with our transcriptional analysis mentioned earlier and the DndD protein was correctly expressed in the complemented strain XTG4/pJTU64 (Fig. 5).

These results suggest that all of the mutations in XTG1–5 are *dnd*-specific and the Dnd proteins are correctly expressed *in vivo*. In the meantime, these expression plasmids carrying individual *dnd *genes could also rescue the Dnd phenotype of other corresponding mutants including HXY1 and HXY2 (data not shown). These complementation results from different mutants further support the conclusion that these mutations are not caused by secondary mutation(s) elsewhere on the chromosome.

### Effect on cell viability by overdosage of Dnd proteins *in vivo*

The above complementation assays for all of the *dnd *mutants were tested without induction by the addition of thiostrepton. Because each individual *dnd *gene is under the control of the thiostrepton-inducible promoter *P*_*tipA *_in the expression plasmids, a feature which could provide us a tool for testing the effect of the over-expressed Dnd protein(s) in cells, we induced Dnd over-expression in the strains XTG1–5 carrying individual *dnd *gene expression plasmids by adding thiostrepton to a final concentration of 5 μg/ml in the normal culture medium. Surprisingly, XTG3/pJTU86 (carrying *dndC*) and XTG4/pJTU64 (carrying *dndD*) completely ceased growth, in sharp contrast to the normal growth of XTG1/pJTU2001 (carrying *dndA*), XTG2/pJTU81 (carrying *dndB*), and XTG5/pJTU65 (carrying *dndE*). This result suggests that over-expression of DndC or DndD proteins *in vivo *has a detrimental effect on cell viability.

## Discussion

Early predictions of genes involved in DNA phosphorothioation and their organization as an operon within a region covering the cloned *dnd *gene cluster was mostly based on bioinformatic analysis, and no detailed experiments had been performed to provide direct evidence. We refined the conclusions by first minimizing the responsible region to a *ca*. 6.7-kb DNA fragment carrying only five genes, which still retained the ability to confer the Dnd phenotype on Dnd^- ^hosts. We went on to confirm the expression of multiple and independent proteins encoded by an operon (*dndB-E*) using systematic mutagenesis either by targeted gene disruption and/or in-frame deletions internal to each protein. We then introduced individual engineered constructs, each containing only one specific gene under the control of a common promoter, into the above mutants. Reversion of the DNA shift from stable to degradation status or *vice versa *demonstrated unambiguously that DndA, C, D, and E are required in the biochemical pathway leading to the Dnd phenotype. The opposite effect of mutation in *dndB *to aggravate the Dnd phenotype was at least partly attributed to the changes of the sequence recognition specificity surrounding the modification sites [8].

The finding that excessive expression of DndC and DndD could affect cell growth and/or viability suggests that the *in vivo *level of the *dnd *system must be tightly regulated, consistent with our earlier observation that not all of the available sites could be modified [8]. This agrees well with the fact that the Dnd phenotype could only be detected when the entire *dnd *gene cluster was integrated into the chromosome or carried on a low copy-number plasmid in ZX1, but not on a plasmid with a copy-number of 10 or more [20]. All of these phenomena suggested that either an unknown mechanism is present in the cell to tightly control DNA phosphorothioation, or that over-expression of some of the proteins to override the regulation could be detrimental to the cells. We propose that the dosage of the Dnd proteins in the cells may not exceed the tolerable limit, and that the Dnd proteins must be balanced so as to be expressed in a highly coordinated manner in the cells. Therefore, simultaneous and/or unbalanced over-expression of one or even all four (*dndB-E*)of the *dnd *genes could seriously harm the cells, leading to inhibition of growth. The present study, showing that strongly induced expression of DndD and DndC, but not the other Dnd proteins, by the addition of thiostrepton, strongly suggests that these two proteins are the key determinants for the phenomenon. Being an IscS-like protein, DndA [21] was suggested to provide sulfur via its L-cysteine desulfurase activity and to catalyze iron-sulfur cluster assembly of DndC [22], probably by generating a persulfide (perhaps with the cysteine residue(s) in DndC or DndD) in the modification process. As such IscS-like proteins are also often required, as multi-functional proteins, for many other metabolic pathways [21], the detrimental effect by over-expression of DndC and DndD could be attributed to deprivation of DndA which is vital for primary metabolism. Thus, the fact that DndA function could not be substituted by other IscS homologs, at least in *S. lividans *analyzed here, might be due to a failure of proper persulfide formation, which could subsequently be delivered to target the DNA via DndC or DndD (not DndE because of its apparent lack of a .cysteine residue mediating persulfide formation). The exact mechanism of negative role of the over-expressed DndC and DndD proteins to cell viability remains, however, to be determined.

## Conclusion

Genetic determination of the Dnd phenotype diagnostic for DNA sulfur modification in *S. lividans *was unambiguously attributed to a 6,665-bp DNA region carrying five *dnd *genes, with *dndB-E *constituting an operon and *dndA *transcribed divergently. Mutations in each of four *dnd *genes (*dndA*, *C*, *D*, and *E*) abolished the Dnd phenotype while mutation of *dndB *aggravated the Dnd phenotype. The Dnd phenotype of all mutants could be restored by complementation with the corresponding *dnd *gene, suggesting that they are essential for DNA sulfur modification. The fact that the cells ceased growth by overdosage of DndC or DndD *in vivo *suggests that the frequency of DNA phosphorothioate modification is under strict control in the native host.

## Methods

### Bacterial strains and plasmids

These are described in Additional file 1.

### Methods and techniques

Standard methods for culturing cells, DNA cloning, PCR, Southern hybridization, and Western blotting were according to [23] in *E. coli *and [24] in *Streptomyces*. PCR primers are listed in Table 1 and PCR products successfully cloned into vectors were confirmed by sequencing. Mutants were confirmed by PCR and Southern hybridization. Tests of Dnd phenotype were described in [5,8] or [10,15].

**Table 1:** primers used in PCR and RT-PCR

Primer Name	Sequence (with the restriction enzyme sites underlined)	Enzyme site
A2	ATCACCCCTTCCACCGAGAT	
A1	ACTGGATGACCGCGGAGTTC	
B1	GAGTACGTTTTTCCGGCCATCC	
B2	TCCTTCAGCGCCTGCTCGAT	
B3	CCAACACCGACTGGGAGGGG	
C1	CAGAGATCGTCGAGGAGCTG	
C2	GATCTTCAACCGCTCGGTGC	
C3	CAGTATCGAACCATGACCCGG	
D1	TGCGGCAAGACGACCCTGCT	
D2	GTCGGCGAGCTGTTCCACCT	
D3	CAGTGATCGACACCCCACTC	
E1	ATGCCGTCTGAGATCACCAT	
E2	ATAAGCAGCGTCTTGCCCAC	
16S rRNA SP	AGTAACACGTGGGCAACTGC	
16S rRNA AP	CTCAGACCAGTGTGGCCGGT	
xtg1	CCGATCTTGTGCCCGCTGATG	
xtg2	GCGC**CTTAAGT**CGTCCCTTGTTC	*Afl*II
xtg3	GAAGGTGTCTT**AGATCT**CCGG	*Bgl*II
xtg4	CTGGCACGACAGGTTTCC	
xtg5	AAGCACCGGTTCAAGACG	*Age*I
xtg6	GCCCAGGTCCGCAAGAA	
xtg7	CTCGTGGTTGAGCGGGACTACGG	
xtg8	CTGGCACCGGTCAAGCCTAGGTG	*Age*I, *Avr*II
xtg9	GGGACAGCCTAGGGGTGATC	*Avr*II
xtg10	ACTGACCGCAGACCGCAAG	
wlr5	CATATGGTGGGATCTTCTGCAGCT	*Nde*I
wlr6	GGATCCTCAATGATGATGATGATGATGTGACTCTCCTCGCAGGTA	*Bam*HI
wlr7	CATATGAGCACCCCCAAGGCG	*Nde*I
wlr11	GGATCCTTAGTGGTGGTGGTGGTGGTGTGCAGGTGCATCGGTGGTGA	*Bam*HI
dnd-1	AGAGATCACCACATATGCACCTGAGCACC	*Nde*I
dnd-2	CAGCCGGATCCTGATCTCAG	*Bam*HI
dndE-L	CACATATGCCGTCTGAGATCACC	*Nde*I
dndE-R	TAAGGCCTATTCGGCGGTGA	

Intensity of DNA bands was quantified from the fluorescence intensity using GeneTool software (Syngene).

### Refinement of the limits of the *dnd *gene cluster

pHZ1900: a 10-kb *Bam*HI fragment from pHZ825 was cloned into pSET152.

pJTU1203 or pJTU1204 (with opposite direction): a 7.9-kb *Mlu*I-*Eco*RI fragment from pHZ1904 was blunt-ended and cloned into the *Eco*RV site of pSET152.

pJTU1208: the 1.0-kb *Bgl*II fragment from pHZ1900 was inserted into the *Bam*HI site of pBluescript II SK (+). Then a 0.3-kb *Sal*I fragment of this plasmid was replaced with a 1.3-kb *Sal*I fragment from pHZ1904 to generate pHZ2850, in which *dndA *accommodated in a 2.0-kb *Bam*HI/*Bgl*II-*Sac*I region. A 1.4-kb fragment from pHZ2850 generated by complete digestion with *Eco*RI and partial digestion with *Bgl*II was inserted into the *Eco*RI and *Bam*HI sites of pSET152 to give pHZ2851. Finally, a 2.1-kb *Xba*I-*Sfi*I fragment of pJTU1204 was replaced with a corresponding 0.8-kb fragment from pHZ2851, generating pJTU1208. Thus, in pJTU1208, the *dnd *gene cluster was shortened to the *Bgl*II site near the end of *dndA*, covering a 6,665-bp region.

pHZ2862 (also the vector for *dndA *deletion): a 2.0-kb *Pvu*II fragment from pHZ1900 was cloned into the *Sma*I site of pBluescript II SK(+) to give pHZ2853, then a 6.5-kb *Sma*I-*Eco*RI fragment from pHZ1900 was used to replace the 0.7-kb corresponding fragment in pHZ2853 to give pHZ2861, in which *dndB-E *lay in a 7.8-kb *Sma*I/*Pvu*II-*Eco*RI region. A 7.8-kb *Bam*HI fragment from pHZ2861 was cloned into pSET152 to give pHZ2862.

### Deletions within *dnd *genes

Vector construction for *dndB *deletion: using pHZ1904 as template, and xtg1 and xtg2 (with introduced *Afl*II site) as primers, a 0.9-kb PCR product was amplified and cloned into pMD18-T (TaKaRa) to generate pJTU1201. Then, the 0.7-kb *Sfi*I-*Afl*II fragment from pJTU1201 was used to replace the 1.4-kb corresponding region in pHZ1904 to result in a *dndB *in-frame deletion vector, pJTU1202, in which a 729-bp DNA fragment was removed from *dndB*.

Vector construction for *dndC *deletion: after pHZ1904 was digested with *Sma*I and *Xba*I, a 5.0-kb fragment carrying *dndC-E *was introduced into the corresponding sites of pUC18 to generate pJTU1205. Using pJTU1205 as template, and xtg3 (with introduced *Bgl*II site) and xtg4 as primers, a 0.9-kb PCR product was amplified and cloned into pMD18-T to give pJTU1209. The 0.5-kb *Afl*II-*Bgl*II fragment from pJTU1209 was used to replace the 1.3-kb corresponding region from pJTU1205 to generate pJTU1210 with an 819-bp in-frame deletion in *dndC*. The 4.8-kb *Afl*II-*Xba*I fragment of pHZ1904 was replaced by the 4.0-kb *Afl*II-*Xba*I fragment of pJTU1210 to generate pJTU1211, which carried *dndC *with an 819-bp in-frame deletion.

Vector construction for *dndD *deletion: using pJTU1205 as template, and xtg5 (with introduced *Age*I site) and xtg6 as primers, a 0.5-kb PCR product was amplified and cloned into pMD18-T to give pJTU1212. The 0.4-kb *Bgl*II-*Age*I fragment from pJTU1212 was used to replace the 2.1-kb corresponding region of pJTU1205 for generation of pJTU1213 with a 1704-bp in-frame deletion in *dndD*. The 4.8-kb *Afl*II-*Xba*I fragment of pHZ1904 was replaced by the 3.1-kb *Afl*II-*Xba*I fragment of pJTU1213 to generat pJTU1214, which carried *dndD *with a 1704-bp in-frame deletion.

Vector construction for *dndE *deletion: using pJTU1205 as template, and xtg7 and xtg8 (with introduced *Age*I and *Avr*II sites) as primers, a 0.7-kb PCR product was amplified and cloned into pMD18-T to give pJTU1215. The 0.6-kb *Age*I-*Mlu*I fragment from pJTU1215 was used to replace a 1.0-kb corresponding region of pJTU1205 to generate pJTU1217 with a 0.4-kb deletion traversing *dndD *and *dndE*. Using pJTU1205 as template, and xtg9 (with introduced *Avr*II site) and xtg10 as primers, a 1.0-kb PCR product was amplified and cloned into pMD18-T to give pJTU1216. The engineered 0.9-kb *Bst*XI-*Avr*II fragment from pJTU1216 was used to replace a 0.7-kb corresponding region of pJTU1217 to generate pJTU1218 with a 216-bp in-frame deletion in *dndE *only. The 4.8-kb *Afl*II-*Xba*I fragment of pHZ1904 was replaced by the 4.6-kb fragment corresponding fragment of pJTU1218 for to generate pJTU1219, which carried *dndE *with 216-bp in-frame deletion.

pHZ2862, pJTU1202, pJTU1211, pJTU1214, pJTU1219 were introduced into HXY6 by conjugation from *E. coli *ET12567 carrying pUZ8002 [25].

### Construction of the expression vectors used in *Streptomyces *each carrying an independent *dnd *gene

*dndA *expression vector: a 1.2-kb engineered *Nde*I-*Bam*HI fragment carrying *dndA *from pHZ882 was inserted into the corresponding sites of pHZ1272 to give pJTU2001.

*dndB *expression vector: using pHZ1904 as template, and wlr5 and wlr6 as primers, a 1.2-kb PCR product carrying *dndB *with introduced *Nde*I and *Bam*HI sites (with C-terminal His-tag) was amplified and cloned into pMD18-T to give pJTU68. Then the corresponding *Nde*I-*Bam*HI DNA fragment from pJTU68 was introduced into pHZ1272 between the restriction sites *Nde*I and *Bam*HI to give pJTU81.

*dndC *expression vector: using pHZ1904 as template, and wlr7 and wlr11 as primers, a 1.5-kb PCR product carrying *dndC *with introduced *Nde*I and *Bam*HI sites (with C-terminal His-tag) was amplified and cloned into pMD18-T to give pJTU72. Then *dndC *from pJTU72 was introduced into pHZ1272 between the restriction sites *Nde*I and *Bam*HI to give pJTU86.

*dndD *expression vector: using pHZ1904 as template, and dnd-1 and dnd-2 as primers, a 2.0-kb PCR product carrying *dndD *with introduced *Nde*I and *Bam*HI sites was amplified, digested with the corresponding enzymes and cloned into pET15b to generate pHZ2893. Then *dndD *from pHZ2893 was introduced into pHZ1272 between the restriction sites *Nde*I and *Bam*HI to give pJTU64.

*dndE *expression vector: using pHZ1904 as template, and dndE-L and dndE-R as primers, a 0.4-kb PCR product carrying *dndE *with introduced *Nde*I site was amplified and cloned into pMD18-T to give pJTU180. Then *dndE *from pJTU180 was introduced into pHZ1272 after digestion with *Nde*I and *Bam*HI to give pJTU65.

### Over-expression and purification of DndD protein

After IPTG induction, *E. coli *BL21 (DE3) containing pHZ2893 over-expressed the DndD fusion protein with a His-tag at the N-terminal end. The fusion protein as inclusion bodies was further purified with an ÄKTA-fast protein liquid chromatography system (FPLC) (Amersham Pharmacia Biotech) and a 5-ml HiTrap chelating column (Amersham Pharmacia Biotech) under denaturing condition. The fusion protein was used for the production of rabbit anti-DndD polyclonal antibody.

### RT-PCR analysis of *dnd *genes

RNA extraction was according to the standard protocol of RNeasy Protect Bacteria Midi Kit from Qiagen Co. Ltd. RT-PCR experiments were performed according to the standard protocol of OneStep RT-PCR Kit from the same company. Primers are listed in Table 1.

## Authors' contributions

TX carried out most of the experiments. JL and ZW performed operon research and constructed *dndA *expression vector in *S. lividans*. Other expression vectors in *S. lividans *were constructed by SC and LW. They also overexpressed and purified DndD for DY to prepare anti-DndD polyclonal antibody. Work on HXY1, 2 was done by XH. pHZ1900 was constructed by AL. Plasmids from pHZ2850 to pHZ2983 were constructed by XZ. ZD oversaw the project. TX, ZW, SC and ZD wrote the paper. All authors discussed the results and assisted with editing of the manuscript.

## Supplementary Material

Additional file 1 **Additional table 1.** Table displaying bacterial strains and plasmids. Click here for file
