# Supplementary material for: DNA phosphorothioation in Streptomyces lividans: mutational analysis of the dnd locus
Source: BMC Microbiol. 2009 Feb 20;9:41. doi: 10.1186/1471-2180-9-41 (PMC2653506; doi:10.1186/1471-2180-9-41)
Supplement: Additional file 1 — Additional table 1. Table displaying bacterial strains and plasmids. [file 1471-2180-9-41-S1.doc]

## Additional table 1 - Bacterial strains and plasmids

| Strain or plasmid | Characteristics^*^ | Source or reference |
| --- | --- | --- |
| *S. lividans* 66 |  |  |
| 1326 | Prototrophic, SLP2^+^, SLP3^+^ |  |
| ZX1 | JT46 derivative with 93-kb deletion carrying a genomic island (SLG) including the *dnd* cluster | [9] |
| LA2 | 1326 derivative with *dndD* disruption | [5] |
| HXY1 | 1326 derivative with *dndA* disruption | [5] |
| HXY2 | 1326 derivative with in-frame deletion of *dndB* | [8] |
| HXY6 | 1326 derivative with complete deletion of *dnd* gene cluster | [8] |
| XTG1 | HXY6 derivative carrying complete *dndB-E* but deletion of *dndA* | This work |
| XTG2 | HXY6 derivative carrying complete *dndA* and *dndC-E* but in-frame deletion of *dndB* | This work |
| XTG3 | HXY6 derivative carrying complete *dndA-B* and *dndD-E* but in-frame deletion of *dndC* | This work |
| XTG4 | HXY6 derivative carrying complete *dndA-C* and *dndE* but in-frame deletion of *dndD* | This work |
| XTG5 | HXY6 derivative carrying complete *dndA-D* but in-frame deletion of *dndE* | This work |
| *E. coli* |  |  |
| DH5α | F^-^, *rec*A, *lac*Z, △M15 | TaKaRa |
| ET12567/pUZ8002 | *Dam*, *dcm*, *hsdS*, pUZ8002 | [25] |
| *E. coli* BL21(DE3) | F^-^, ompT, hsdS_B_ (r_B_^−^ m_B_^−^), gal, dcm, (DE3) | Promega |
| Plasmids |  |  |
| pBluescript II SK(+) | *E. coli* cloning vector, *bla* | Fermentas |
| pET15b | *E. coli* expression vector | Novagen |
| pMD18-T | *E. coli* vector for DNA sequencing | TaKaRa |
| pUC18 | *E. coli* cloning vector, *bla* | Fermentas |
| pSET152 | *lacZ, rep^puc^, att^ФC31^, oriT* | [17] |
| pHZ825 | SuperCos1 derivative carrying a 12-kb *Eco*RI fragment including the entire *dnd* gene cluster | [5] |
| pHZ209 | pIJ101 derivative, tsr, oriT, ori^pIJ101^ | [8] |
| pHZ882 | pET15b derivative with an engineered 1.2-kb *Nde*I-*Bam*HI fragment carrying *dndA* | [22] |
| pHZ1272 | *Streptomyces* expression vector with the thiostrepton-inducible promoter *P_tipA_*, *neo, tsr* | [18] |
| pHZ1900 | pSET152 derivative carrying a *c.* 9.7-kb *Bam*HI fragment carrying the complete *dnd* gene cluster | Fig. 1 |
| pHZ1904 | pSET152 derivative carrying a *c.* 8.0-kb DNA fragment with the complete *dnd* gene cluster | [5]; Fig. 1 |
| pHZ2850 | pBluescript II SK(+) derivative with a 2.0-kb *Sac*I-*Bgl*II fragment carrying *dndA* cloned into its *Bam*HI-*Sac*I sites | This work |
| pHZ2851 | pSET152 derivative with a 1.4-kb DNA fragment carrying *dndA* lifted from pHZ2850 as a *Bgl*II-*Eco*RI fragment for cloning into its *Bam*HI-*Eco*RI sites | This work |
| pHZ2853 | pBluescript II SK(+) derivative with a 2.0-kb *Pvu*II fragment carrying *dndB* from pHZ1900 cloned into its *Sma*I site | This work |
| pHZ2861 | pBluescript II SK(+) derivative with a 7.8-kb *Pvu*II-*Eco*RI fragment carrying *dndB-E* from pHZ1900 cloned into its *Sma*I-*Eco*RI sites | This work |
| pHZ2862 | pSET152 derivative carrying the *dnd* gene cluster with deletion of 651 bp in the 3’ terminus of *dndA* | This work, |
| pHZ2893 | pET15b derivative with a 2.0 kb engineered *Nde*I-*Bam*HI fragment carrying *dndD* | This work |
| pJTU64 | pHZ1272 derivative carrying *dndD* as a 2.0-kb *Nde*I-*Bam*HI fragment lifted from pHZ2893 | This work |
| pJTU65 | pHZ1272 derivative carrying *dndE* as a 0.4 kb *Nde*I-*Bam*HI fragment lifted from pJTU180 | This work |
| pJTU68 | pMD 18-T derivative with a 1.2-kb engineered *Nde*I-*Bam*HI fragment carrying *dndB* with a C-terminal His-tag | This work |
| pJTU72 | pMD 18-T derivative with a 1.5 kb engineered *Nde*I-*Bam*HI fragment carrying *dndC* with a C-terminal His-tag | This work |
| pJTU81 | pHZ1272 derivative carrying *dndB* with a C-terminal His-tag as a 1.2-kb *Nde*I-*Bam*HI fragment lifted from pJTU68 | This work |
| pJTU86 | pHZ1272 derivative carrying *dndC* with a C-terminal His-tag as a 1.5-kb *Nde*I-*Bam*HI fragment lifted from pJTU72 | This work |
| pJTU180 | pMD 18-T derivative carrying an engineered 0.4-kb *dndE* structural gene fragment with an introduced *Nde*I site | This work |
| pJTU1201 | pMD 18-T derivative carrying a 0.9-kb PCR product with introduced *Afl*II site | This work |
| pJTU1202 | pSET152 derivative carrying the *dnd* gene cluster with a 729-bp deletion internal to *dndB* | This work |
| pJTU1203 (1204) | pSET152 derivative, a 7.9-kb blunted-ended *Mlu*I-*Eco*RI fragment from pHZ1904 carrying the complete *dnd* gene cluster inserted into the *Eco*RV site (with pJTU1204 in opposite direction) | Fig. 1 |
| pJTU1205 | pUC18 derivative carrying a 5.0-kb *Sma*I-*Xba*I fragment from pHZ1904 | This work |
| pJTU1208 | pSET152 derivative carrying the 6.6-kb *dnd* gene cluster | Fig. 1 |
| pJTU1209 | pMD 18-T derivative carrying a 0.9-kb PCR product with introduced *Bgl*II site | This work |
| pJTU1210 | pJTU1205 derivative with a 819-bp deletion internal to *dndC* | This work |
| pJTU1211 | pSET152 derivative carrying the *dnd* gene cluster with an internal 819-bp deletion in *dndC* | This work |
| pJTU1212 | pMD 18-T derivative carrying a 0.5-kb PCR product with introduced *Age*I site | This work |
| pJTU1213 | pJTU1205 derivative with an internal 1,704-bp deletion in *dndD* | This work |
| pJTU1214 | pSET152 derivative carrying the *dnd* gene cluster with an internal 1,704-bp deletion in *dndD* | This work |
| pJTU1215 | pMD 18-T derivative carrying a 0.6-kb PCR product with introduced *Avr*II and *Age*I sites | This work |
| pJTU1216 | pMD 18-T derivative carrying a 1.0-kb PCR product with introduced *Avr*II site | This work |
| pJTU1217 | pJTU1205 derivative with a 0.4-kb deletion in *dndD* and *dndE* | This work |
| pJTU1218 | pJTU1205 derivative with an internal 216-bp deletion in *dndE* | This work |
| pJTU1219 | pSET152 derivative carrying the *dnd* gene cluster with an internal 216-bp deletion in *dndE* | This work |
| pJTU2001 | pHZ1272 derivative carrying *dndA* as a *Nde*I-*Bam*HI fragment from pHZ882 | This work |

** oriT*, origin of transfer of plasmid RK2; *aadA,* *bla, neo,* and *tsr* denote genes encoding resistance to spectinomycin/streptomycin, ampicillin, kanamycin, and thiostrepton, respectively
